# Solution Process Synthesis of High Aspect Ratio ZnO Nanorods on Electrode Surface for Sensitive Electrochemical Detection of Uric Acid

**DOI:** 10.1038/srep46475

**Published:** 2017-04-18

**Authors:** Rafiq Ahmad, Nirmalya Tripathy, Min-Sang Ahn, Yoon-Bong Hahn

**Affiliations:** 1 School of Semiconductor and Chemical Engineering, Nanomaterials Processing Research Center, Chonbuk National University, 567 Baekjedaero, Deokjin-gu, Jeonju-si, Jeollabuk-do, 54896, Republic of Korea; 2 Department of BIN Fusion Technology, Chonbuk National University, 567 Baekjedaero, Deokjin-gu, Jeonju-si, Jeollabuk-do, 54896, Republic of Korea

## Abstract

This study demonstrates a highly stable, selective and sensitive uric acid (UA) biosensor based on high aspect ratio zinc oxide nanorods (ZNRs) vertical grown on electrode surface via a simple one-step low temperature solution route. Uricase enzyme was immobilized on the ZNRs followed by Nafion covering to fabricate UA sensing electrodes (Nafion/Uricase-ZNRs/Ag). The fabricated electrodes showed enhanced performance with attractive analytical response, such as a high sensitivity of 239.67 μA cm^−2^ mM^−1^ in wide-linear range (0.01–4.56 mM), rapid response time (~3 s), low detection limit (5 nM), and low value of apparent Michaelis-Menten constant (*K*_m_^app^, 0.025 mM). In addition, selectivity, reproducibility and long-term storage stability of biosensor was also demonstrated. These results can be attributed to the high aspect ratio of vertically grown ZNRs which provides high surface area leading to enhanced enzyme immobilization, high electrocatalytic activity, and direct electron transfer during electrochemical detection of UA. We expect that this biosensor platform will be advantageous to fabricate ultrasensitive, robust, low-cost sensing device for numerous analyte detection.

Abnormal uric acid (UA) levels in biological fluids affect millions of people worldwide causing several disorders such as gout, uric acid kidney stones, cardiovascular and renal diseases, hypertension, obesity, fatty liver, metabolic syndrome, and diabetes^1^,^2^. Thus, there is a high need to test UA levels routinely for better disease screening, monitoring and treatment. However, most of the methods such as spectrophotometry, ion chromatography, high-performance liquid chromatography (HPLC) mass spectrometry, chemiluminescence, capillary electrophoresis-amperometry, colorimetry, and enzymatic test-kits used for UA determination are expensive, complex, time consuming, and laborious^3^,^4^,^5^,^6^,^7^,^8^,^9^.

The electrochemical based methods for UA sensing offers a great potential for rapid, reliable, easy to use, low cost and portable devices for routine analysis^10^,^11^,^12^,^13^,^14^,^15^,^16^,^17^,^18^,^19^. Recently, varieties of nanostructured materials i.e. zinc oxide (ZnO), copper oxide (CuO), iron oxide (Fe_2_O_3_), cerium oxide (CeO_2_), tin oxide (SnO_2_), zirconium oxide (ZrO_2_), titanium oxide (TiO_2_), and magnesium oxide (MgO), etc. have shown great potential for different sensing devices fabrication^20^,^21^,^22^,^23^,^24^,^25^. Among them, nanostructured ZnO with band gap of 3.37 eV is an excellent candidate to be used as matrix for biosensor fabrication due to its simple and cost-effective synthesis, high surface area, low toxicity, good chemical stability and biological compatibility, and high electron mobility^26^,^27^,^28^. Importantly, high isoelectric point (IEP = ~9.5) of ZnO makes it suitable for absorption of low IEPs proteins or enzymes such as uricase (IEP = ~4.64) at physiological pH, because the enzyme immobilization is primarily driven by electrostatic interaction^29^. A comparative study of previously reported ZnO nanostructure based UA biosensors are listed in Table 1. These electrochemical methods detected the UA, however most of them fabricated UA sensing devices using conventional methods (separately synthesized nanostructure, mixed with binder and then casted on electrode surface), which have shown poor sensitivity, stability and reproducibility. Thus, direct growth of nanostructures on electrode surface is needed to keep their morphology, so that same kind of nanostructure can be available in the applications. Also, directly grown nanostructures will not only provide stable electrodes but also large specific surface area for abundant enzyme loadings.

In this work, uniform vertical grown ZnO NRs with high aspect ratio were synthesized via a simple one-step low temperature solution route on a ZnO seeded silver (Ag) electrode surface. The uricase enzyme was immobilized on the ZnO NRs/Ag electrode to fabricate UA sensing device. The biosensor showed enhanced sensing performance for UA detection which can be attributed to the high surface area of vertical grown ZnO NRs leading to abundant enzyme immobilization and direct electron transfer. This sensing system was designed to enable the fabrication of robust, ultrasensitive and highly reproducible UA biosensor device.

## Results and Discussions

### Characterization of the ZNR

**Figure 1:**
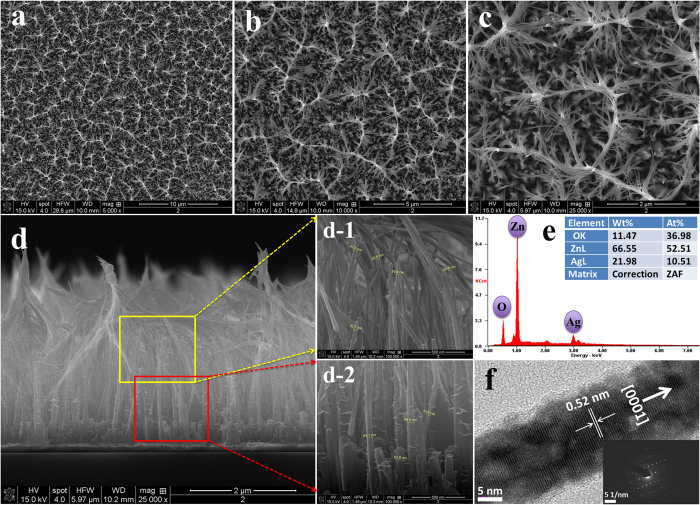
(**a–c**) Typical field emission scanning electron microscopy (FESEM) images showing top-view at different magnifications, and (**d**) low resolution cross-sectional view with magnified views (d-1, d-2), which indicate that the ZNRs were vertically grown and have an average length of ~3.6 μm and an average top and bottom diameter of ~30 nm and ~60 nm, respectively; (**e**) Energy-dispersive X-ray spectra of as-grown ZNRs on Ag electrode surface; (**f**) high-resolution transmission electron microscopy (HRTEM) image showing the lattice fringes of ZnO. Inset f shows the selected area electron diffraction (SAED) pattern of ZnO that suggests the ZNR growth is along the [0001] direction.

**Figure 2:**
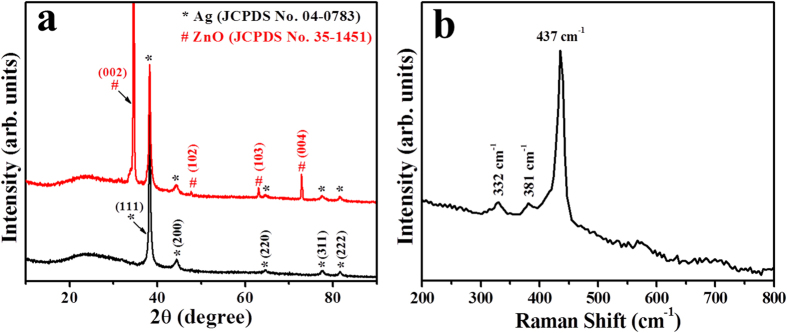
(**a**) X-ray diffraction pattern of bare Ag electrode (black line) and ZNRs grown on Ag electrode and (**b**) Raman-scattering spectra of ZNRs.

The surface morphology of the as-synthesized ZNRs was characterized by field emission scanning electron microscopy (FESEM) and high-resolution transmission electron microscopy (HRTEM) technique, the obtained images are presented in Fig. 1. Figure 1a–c show the low and high resolution FESEM images depicting the surface morphology of ZNRs grown on Ag electrode surface. It can be clearly seen that ZNRs were uniformly grown and they were attached to each other at the top due to their high vertical length. The vertical growth was further confirmed by cross-sectional analysis of ZNRs grown on Ag electrode (Fig. 1d) which shows ZNRs were relatively well-grown on Ag electrode with ~3.6 μm average length of ZNRs. High resolution FESEM images at the top (Fig. 1d-1) and at bottom (Fig. 1d-2) of ZNRs show the average diameter of ~30 nm and ~60 nm, respectively. Further, the aspect ratio of ZNRs was calculated by taking the ratios of average length and average diameter (L/D), which shows as-synthesized ZNRs were of high aspect ratio (~80) that is significantly higher for solution based synthesis method. The chemical composition of as-synthesized ZNRs was measured via energy-dispersive X-ray spectra (EDX), shown in Fig. 1e. The spectra show the presence of zinc (Zn), oxygen (O), and Ag peaks only, where Ag is attributed to the Ag electrode. There was no other peak found in the spectrum due to absence of impurities on the ZNRs/Ag electrode surface.

The surface morphology and crystallites of single ZNR was investigated by high-resolution transmission electron microscopy (HRTEM) analysis. Figure 1f shows the HRTEM image of single ZNR with interplanar spacing of about 0.52 nm, which corresponds to the (0001) planes of ZnO. Together, HRTEM image and selected area electron diffraction (SAED) pattern (see inset of Fig. 1f) confirms the single crystalline nature of ZnO with the wurtzite hexagonal phase and preferential growth along the [0001] direction.

We also examined the structural and chemical properties of as-synthesized ZNRs by X-ray diffraction (XRD) and Raman spectroscopy techniques (Fig. 2). Figure 2a shows the XRD pattern for bare Ag electrode (black line) and ZNRs grown on Ag electrode (red line). From the XRD spectrum of bare Ag electrode displays the peaks at 38.3 and 44.4° which can be indexed to (111) and (200) planes of pure silver (JCPDS card No. 04–0783). From the XRD spectrum of ZNRs grown on Ag electrode, a clear and strong peak (002) indicates the growth direction of ZNR is along c-axis which is indexed to the wurtzite-structured hexagonal phase of single crystalline bulk ZnO with lattice constant of a = 3.249 and c = 5.206 Å (JCPDS card No. 35–1451), in addition to the above Ag electrode peaks. This further confirms the directly grown ZNRs on the surface of Ag electrode. Raman spectra (Fig. 2b) further confirm the crystalline nature of ZNRs which is dominated by three peaks at about 332, 381, and 437 cm^−1^. The dominant and sharp peak at 437 cm^−1^ is assigned to the Raman active optical phonon E_2_ (high) mode for the wurtzite ZnO, and the two weak peaks at 332 and 381 cm^−1^ assigned to E_2H_-E_2L_ (multi phonon process) and A_1T_ modes, respectively^30^. No additional peaks were found in the XRD and Raman spectra, thus confirms the good structural and chemical properties (i.e. growth orientation, purity, and crystallinity) of as-synthesized ZNRs.

### Electrochemical properties of fabricated electrodes and amperometric detection of UA

**Figure 3:**
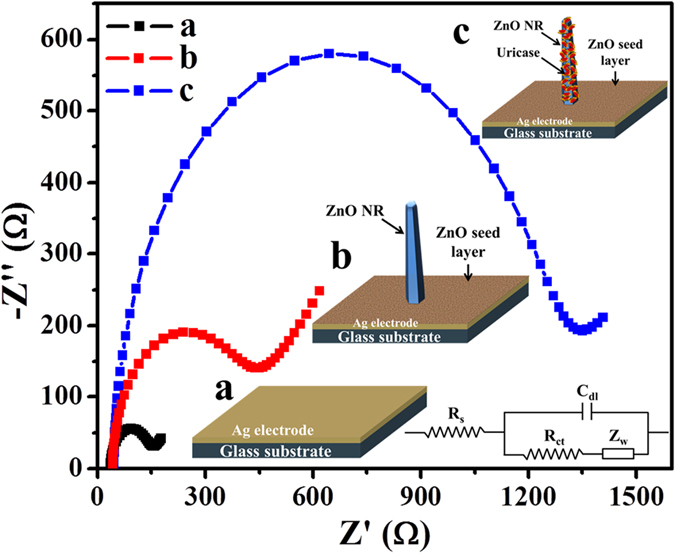
Typical Nyquist plots of the imaginary part of the impedance (Z’) vs. the real part of the impedance (Z”) for (**a**) Ag/glass, (**b**) ZNRs/Ag/glass, and (**c**) Nafion/Uricase-ZNRs/Ag/glass electrodes within a frequency range from 0.1 Hz to 100 MHz with applied amplitude of ± 5 mV in PBS containing 5 mM Fe(CN)6^3−/4−^ and 0.1 M KCl. Inset show the schematic of different electrodes (**a–c**) and Randle equivalent circuit model, which was obtained by parameters like solution resistance (R_s_) is in series with charge transfer resistance (R_ct_) in parallel with double-layer capacitance (C_dl_).

The electrochemical impedance spectroscopy (EIS) measurements of the Ag/glass, ZNRs/Ag/glass, and Nafion/Uricase-ZNRs/Ag/glass electrodes were measured to study the effective fabrication process of sensing electrodes (Fig. 3). The EIS is a well-known technique to study the changes of electrode surface state and charge transfer properties of the electrode^31^. For EIS spectra measurement, an electron transfer probe [Fe(CN)6^3−/4−^] was used for electrode surface characterization. After measurement, the obtained data was fitted with a Randle equivalent circuit model (inset of Fig. 3), where the parameters like solution resistance (R_s_) is in series with charge transfer resistance (R_ct_) in parallel with double-layer capacitance (C_dl_). The semicircle diameter of the nyquist plot equals to the charge transfer resistance (R_ct_) which indicates the charge transfer kinetics of the redox probe at the electrode surface. From Fig. 3, the EIS spectrum of Ag/glass electrode (a) exhibited low R_ct_ value with good charge transfer rate due to high conductive nature of Ag electrode. However, post-seed layer deposition and growth of ZNRs (b), the Nyquist semicircle becomes larger which may be due to less conductivity of ZnO that hinders the electron transfer. Then after uricase enzyme immobilization, the R_ct_ values of Nafion/Uricase-ZNRs/Ag/glass electrodes (c) was further increased due to non-conductive uricase, indicating the successful immobilization of enzyme that forms a physical barrier between redox probe [Fe(CN)6]^3−/4−^ and the Ag electrode surface.

**Figure 4:**
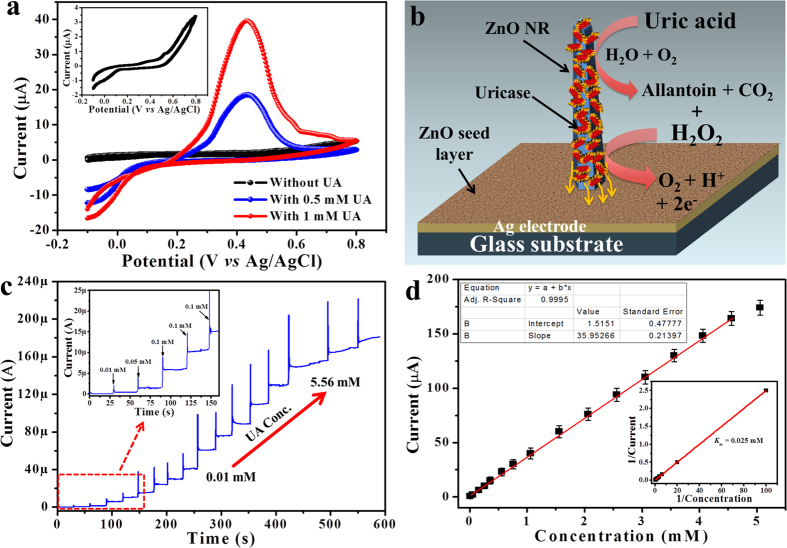
Electrochemical detection of UA. (**a**) Cyclic voltammetry (CV) response of Nafion/Uricase-ZNRs/Ag electrode in 0.05 M PBS solution (pH 7.0) without (black line) and with 0.5 and 1.0 mM UA recorded with a scan rate of 100 mV/s in the potential range of −0.1- +0.8 V. Inset shows the CV response in only 0.05 M PBS solution (without UA); (**b**) Schematic illustration of sensing device showing UA sensing mechanism; (**c**) Amperometric responses of the Nafion/Uricase-ZNRs/Ag electrode under successive addition of UA in 0.05 M PBS solution (pH 7.0) in the total concentration range from 0.01 to 5.56 mM at an applied potential of +0.42 V *vs.* Ag/AgCl. Inset shows the enlarged amperometric responses with low UA concentration; (**d**) the corresponding current-UA concentration calibration curve. Inset shows the Lineweaver-Burk plot of 1/i *vs.* 1/C.

Further, the electrochemical properties of fabricated sensor electrode (Nafion/Uricase-ZNRs/Ag/glass) was characterized by cyclic voltammetry (CV) method in 0.05 M PBS solution (pH 7.0) between the potentials of −0.1 and +0.8 V at a scan rate of 100 mV/s. As shown in the Fig. 4a, in UA absence (black line) the Nafion/Uricase-ZNRs/Ag/glass electrode didn’t show any obvious current peak (inset of Fig. 4a). However, when the CV was measured in the presence of 0.5 and 1.0 mM UA, a broad and clear oxidation peak with peak potential of about +0.42 V was observed due to the electrocatalytic oxidation of UA. The electro-oxidation mechanism of UA sensing along with device schematic is illustrated in Fig. 4b. During electrocatalytic oxidation, the UA was oxidized to allantoin and produce CO__2_ _+_ _H_2_O_2_. The produced H_2_O_2_ further generate electrons during its oxidation through the working electrode and hence enhance the current response^32^,^33^. The enhanced response can be attributed to the high aspect ratio of ZNRs that provide a very high specific surface area for enough enzyme loading on vertically grown ZNRs. The vertically grown ZNRs on electrode surface also facilitate fast and direct electron transfer between the active sites of immobilized uricase and the Ag electrode surface.

The amperometric response of the fabricated electrodes (Nafion/Uricase-ZNRs/Ag) were recorded to determine the performance for UA sensing application by successively adding UA in an increasing concentration range of 0.01 to 5.56 mM, in 0.05 M PBS solution (pH 7.0) at an applied potential of +0.42 V (*vs.* Ag/AgCl) under stirring condition and shown in Fig. 4c. The Nafion/Uricase-ZNRs/Ag electrodes showed excellent amperometric response with successive addition of certain amount of UA in 0.05 M PBS solution (pH 7.0). From the graph, a clear steady current increase is evident with an increasing addition of UA i.e. 0.01 to 5.56 mM. Further, a rapid, sensitive, stable, and well-defined amperometric response presented a short response time of ~3 s for the sensing electrode to reach steady state current. The inset in Fig. 4c shows the enlarged amperometric response at low concentration range from 0.01 to 0.36 mM. The calibration curve of corresponding amperometric response of Nafion/Uricase-ZNRs/Ag electrode is shown in Fig. 4d, it can be seen from the plot that the current response increases with increasing UA concentration. However, at higher UA concentration increase in current was saturated, which suggests the saturation of active sites of the uricase enzymes at those UA concentration levels. From the calibration plot, a clear and wide linear range from 0.01 to 4.56 mM was obtained with high linear relationship between UA concentration and current response (correlation coefficient (R^2^) = 0.9995). A linear equation was obtained from the graph; y = 35.95266x + 1.5151 (R^2^ = 0.9995), where y and x stand for the current (μA) and the concentration (mM) of UA, respectively. From the slope of linear portion of calibration curve, the high sensitivity of 239.67 μA cm^−2^ mM^−1^ in wide linear range (0.01–4.56 mM) was obtained, which is the highest value reported for UA sensing using other ZnO nanostructures (Table 1). Also, the low detection limit (LOD) was estimated to be 5 nM (based on S/N ratio) for the fabricated sensor electrode. The inset of Fig. 4d shows the Lineweaver-Burk plot (1/i *vs.* 1/C) used to calculate apparent Michaelis-Menten constant (K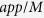) from the Lineweaver-Burk equation 1/i = (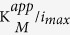) (1/C) + 1/*i*_*max*_), where i is the current, *i*_*max*_ is the maximum current measured under saturated condition, and C is the UA concentration. The low 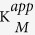 value of 0.025 mM confirms the higher affinity of enzymes for UA detection. The high sensing performance of electrode can be ascribed to the high specific surface area due to high aspect ratios of vertically grown ZNRs on electrode surface, which provides a favorable microenvironment for uricase loading in large quantity. The direct electron also leads to the higher-sensitivity, fast response and lower detection limit.

### Anti-interference, reproducibility, and stability test of electrodes

**Figure 5:**
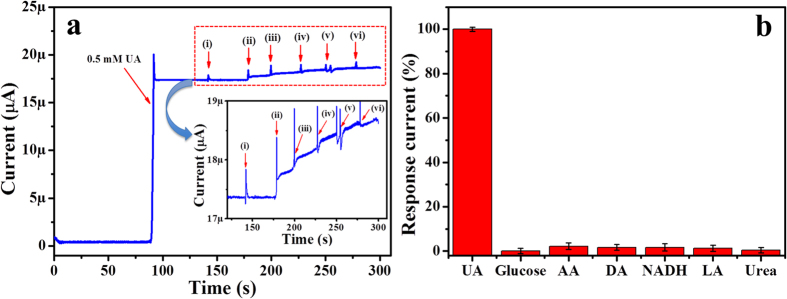
Anti-interference property of UA sensing electrode. (**a**) amperometric response of Nafion/Uricase-ZNRs/Ag electrode in 0.05 M PBS solution (pH 7.0) at an applied potential of +0.42 V *vs.* Ag/AgCl with successive addition of 0.5 mM UA and 100 μM of each interfering species i.e. (i) glucose, (ii) ascorbic acid (AA), (iii) dopamine (DA), (iv) nicotinamide adenine dinucleotide (NADH), (v) lactic acid (LA), and (vi) urea; (**b**) the bar chart shows the corresponding calibrated response with addition of UA and interfering species.

The anti-interference, reproducibility, and stability test of the Nafion/Uricase-ZNRs/Ag electrodes were further evaluated. The anti-interference properties are important parameters for electrochemical based sensing, as the working potential may be related to the oxidation of other potential interfering species present with UA. Also, the better selectivity will ensure the high accuracy during measurement. Herein, we carried out interference test by successive addition of 0.5 mM UA, and 100 μM of each interfering species i.e. glucose, AA, DA, NADH, LA, and urea (Fig. 5a). From the figure, the addition of 0.5 mM UA resulted in significant and quick current response, whereas addition of interfering species caused negligible current changes. A magnified view of amperometric response for interfering species addition is shown in the inset of Fig. 5a. Compared current response for UA detection with other interfering species was illustrated by histogram in Fig. 5b. It can be seen from graph that the interfering species caused very low response i.e. for 100 μM glucose (~.3%), for 100 μM AA (~2.3%), for 100 μM DA (~2.4%), for 100 μM NADH (~1.7%), for 100 μM LA (~1.8%), and for 100 μM urea (~0.5).

**Figure 6:**
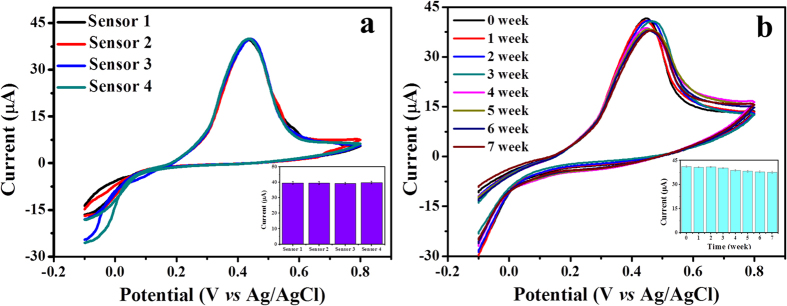
Reproducibility and stability measurements. (**a**) CV response of five Nafion/Uricase-ZNRs/Ag electrodes prepared in similar conditions for the detection of 1.0 mM UA in 0.05 M PBS solution (pH 7.0). Inset shows the calibrated histogram; (**b**) the CV response of Nafion/Uricase-ZNRs/Ag electrodes for the detection of 1.0 mM UA in 0.05 M PBS solution (pH 7.0) for seven weeks. Inset shows the calibrated histogram of stability test.

Further, to evaluate the reproducibility of Nafion/Uricase-ZNRs/Ag electrodes, we fabricated four sensing electrodes in similar conditions and used them for the detection of 1.0 mM UA in 0.05 M PBS solution (pH 7.0) by CV, and shown in Fig. 6a. The calibrated histogram is shown in the inset of Fig. 6a which showed a good relative standard deviation (RSD) of ~1%. The repeatability of the fabricated electrode was also measured for ten times and a RSD of 2.6% was obtained. Additionally, the long-term stability of the Nafion/Uricase-ZNRs/Ag electrode was also assessed by storing the fabricated electrode at 4 °C and regular performance/response evaluation for the detection of 1.0 mM UA in 0.05 M PBS solution (pH 7.0) by CV (Fig. 6b). The histogram in the inset of Fig. 6b shows the calibrated CV response which showed good stability as the decrease in sensitivity of the electrode was around ~8% after seven weeks of storage. Together with good anti-interference, reproducibility and stability, the present UA sensing electrode was quite reliable for UA detection.

## Conclusions

In conclusion, we demonstrate the fabrication of stable and highly sensitive UA biosensor based on uniform vertical grown ZnO NRs with high aspect ratio via a simple one-step low temperature solution route. The performance of fabricated biosensor electrode was investigated by measuring the amperometric response with successive addition of different UA concentrations. The results showed high sensitivity (239.67 μA cm^−2^ mM^−1^) in wide linear range (0.01–4.56 mM), fast response time (~3 s), low detection limit (5 nM) and *K*_m_^app^ value (0.025 mM, excellent selectivity, good reproducibility, and long-term storage stability. The obtained results can be attributed to the high aspect ratio of vertically grown ZNRs which provides high surface area leading to enhanced enzyme immobilization. As a result, high electrocatalytic activity of uricase enzyme on ZNRs favored the direct electron transfer between the enzymes active site and ZNRs surface, and transport of electrons from ZNRs to the bottom Ag electrode. Furthermore, direct growth of nanostructures on electrode surface provides a simple yet robust biosensor fabrication platform that would be helpful for the future design of low-cost biosensors electrodes with high performance.

## Methods

### Materials

All chemicals were analytical grade and used without further purification. Zinc nitrate hexahydrate [Zn(NO_3_)_2_·6H_2_O, 99%], hexamethylenetetramine [HMTA; C_6_H_12_N_4_, 99%], uricase (EC 1.7.3.3, from *Arthrobacter gloiformis*), Nafion (5 wt.% in lower aliphatic alcohol and water mixture), uric acid (UA), glucose (D-(+)−99.5%), ascorbic acid (AA), dopamine (DA), nicotinamide adenine dinucleotide (NADH), lactic acid (LA), urea, sodium phosphate dibasic dihydrate (Na_2_HPO_4_·2H_2_O), sodium phosphate monobasic anhydrous (NaH_2_PO_4_), and sodium chloride (NaCl) were purchased from Sigma-Aldrich. Phosphate buffer saline solution (PBS; 0.05 M, pH 7.0) was prepared by mixing solutions of NaH_2_PO_4_, Na_2_HPO_4_·2H_2_O, and NaCl (0.9%) in deionized water. Enzyme solution was prepared by dissolving 1 mg/mL uricase in PBS solution.

### Preparation of high aspect ratio ZNRs/Ag electrodes

To synthesize high aspect ratio ZNRs on Ag electrode surface, first, glass substrates (2.5 cm × 0.3 cm) were cleaned using detergent, diluted water, acetone, and ethanol, subsequently. Before Ag deposition, glass substrates were plasma treated to improve the adhesion property of the substrates. Then, a thin film of Ag (~150 nm) was sputtered on glass substrate followed by ZnO seed layer (~50 nm) deposition on the Ag electrode (0.5 cm × 0.3 cm, rest of the electrode area was covered) using ZnO powder as a sputtering target. Next, above seeded electrodes were suspended upside down using a Teflon sample holder in Pyrex glass bottle containing 50 mL distilled water with an equal molar solutions of Zn(NO_3_)2·6H_2_O (0.03 M) and HMTA (0.03 M). Then the growth process was completed inside a laboratory oven at 90 °C for 16 h. After the hydrothermal reaction, the ZNRs/Ag electrodes were rinsed with isopropanol and distilled water to remove the impurities before characterizations and further application.

### Fabrication of UA sensing electrodes

Before enzyme immobilization on the prepared ZNRs/Ag electrodes, the electrodes were treated with PBS and dried by high purity nitrogen gas to generate hydrophilic surfaces on ZNRs. The direct immobilization of uricase onto ZNRs/Ag electrodes was achieved by keeping 20 μL of uricase for 12 h at 4 °C followed by washing the electrodes with PBS (pH 7.0) to remove the loosely bound enzyme and air-dried. After that, 2 μL of 0.5 wt% Nafion solution was applied to the electrode surface to eliminate the possible fouling and prevent the leaching of the enzyme. As-prepared electrodes (Nafion/Uricase-ZNRs/Ag) were stored in dry condition at 4 °C when not in use.

### Materials characterization

The electrodes morphologies were examined by field emission scanning electron microscopy (FESEM, Hitachi S4700, and SUPRA 40VP) equipped with an energy dispersive X-ray (EDX), and high-resolution transmission electron microscopy (HRTEM, JEOL JEM-2010UHR) with selected area electron diffraction (SAED) at an acceleration voltage of 200 kV. The crystalline structure of as-synthesized ZNRs was analyzed using X-ray diffractometer (XRD, Rigaku) with Cu-Kα radiation (λ = 1.54178 Ǻ) in the range of 10–90° with 8°/min scanning speed. In addition, the optical properties of ZNRs were characterized by Raman-scattering measurements with Ar^+^ (513.4 nm) as the exciton source.

### Electrochemical measurements

All the electrochemical measurements i.e. cyclic voltammetry (CV) and amperometry were performed using an electrochemical measurement station (Ivium CompactStat.e; Ivium Technologies) connected to computer with a conventional three-electrode configuration: a working electrode (Nafion/Uricase-ZNRs/Ag), platinum (Pt) wire as counter electrode, and Ag/AgCl (saturated with KCl solution) as reference electrode.

### Impedance spectroscopy measurements

The electrochemical impedance spectroscopy (EIS) measurements were carried out in a mixture of 5 mM [Fe(CN)6]^3−/4−^ and 0.1 M KCl solutions using an electrochemical measurement station (Ivium CompactStat.e; Ivium Technologies). To characterize the different electrodes, for each electrode the potentiostatic EIS was taken within a frequency range from 0.1 Hz to 100 MHz with applied amplitude of ± 5 mV. All the measurements were conducted at room temperature.

## Additional Information

**How to cite this article**: Ahmad, R. *et al*. Solution Process Synthesis of High Aspect Ratio ZnO Nanorods on Electrode Surface for Sensitive Electrochemical Detection of Uric Acid. *Sci. Rep.* **7**, 46475; doi: 10.1038/srep46475 (2017).

**Publisher's note:** Springer Nature remains neutral with regard to jurisdictional claims in published maps and institutional affiliations.

## Figures and Tables

**Table 1:** Comparison of the Nafion/Uricase-ZNRs/Ag/glass electrodes with other UA sensors.

Working electrode	Sensitivity	LOD (μM)	Linear range (mM)	Ref.
Nafion/Uricase/ZnO NSs/Ag/Si	129.81 μA mM^^−1^^cm^^−2^^	0.019	0.05–2.0	33
Nafion/Uricase/ZnO nanotubes/Au	~68 mV/decade	0.0005	0.0005–1.5	34
Nafion/Uricase/ZnO NRs/GCE	—	0.002	0.005–1.0	35
Uricase/Au NPs/MWCNT/Au	0.44 mA mM^−1^	—	0.10–0.8	36
Nafion/Uricase/ZnO NWs/Au	32 mV/decade	—	0.001–1.0	37
Nafion/Uricase/ZnO NFs/Au	~66 mV/ decade	0.5	0.0005–1.5	38
Nafion/Uricase/ZnO micro/NWs/Au	89.74 μA mM^^−1^^cm^^−2^^	25.6	0.1–0.59	39
Uricase/ZnO:N/ITO/glass	1100 μA mM^^−1^^cm^^−2^^	—	0.05–1.0	40
Nafion/Uricase-ZnO NRs/Ag/glass	239.67 μA mM^^−1^^cm^^−2^^	0.005	0.01–4.56	This work
